# ERRATUM: Pattern of nursing interventions performed on trauma victims according to the Nursing Activities Score

**DOI:** 10.1590/1980-220X-REEUSP-2024-ER05en

**Published:** 2024-09-23

In the article “Pattern of nursing interventions performed on trauma victims according to the Nursing Activities Score”, with the DOI: https://doi.org/10.1590/S0080-623420150000700005, published by the journal: “Revista da Escola de Enfermagem da USP, volume 49(Esp) of 2015, p. 28–34, on page 34:

**Where it was written:**

## Financial support

Fundação de Amparo à Pesquisa do Estado de São Paulo – FAPESP. Process number: 2009/50355-4.

**Now read:**

## Financial support

Fundação de Amparo à Pesquisa do Estado de São Paulo – FAPESP. Process number: 2009/50355-4. This study was financed in part by the Coordenação de Aperfeiçoamento de Pessoal de Nível Superior – Brasil (CAPES) – Finance Code 001.

