# Supplementary material for: ERRATUM: Pattern of nursing interventions performed on trauma victims according to the Nursing Activities Score
Source: Rev Esc Enferm USP. 2024 Sep 23;58:e2024ER05. doi: 10.1590/1980-220X-REEUSP-2024-ER05en (PMC11421567; doi:10.1590/1980-220X-REEUSP-2024-ER05en)
Supplement: Translation

# Translation: ERRATA: Padrão de intervenções de enfermagem realizadas em vítimas de trauma segundo o Nursing Activities Score

**DOI:** 10.1590/1980-220X-REEUSP-2024-ER05pt

No artigo "Padrão de intervenções de enfermagem realizadas em vítimas de trauma segundo o Nursing Activities Score", com número de DOI: https://doi.org/10.1590/S0080-623420150000700005, publicado na Revista da Escola de Enfermagem da USP, volume 49(Esp) de 2015, p. 29-35, na página 35:

**Onde se lia:**

## Apoio financeiro

Fundação de Amparo à Pesquisa do Estado de São Paulo (FAPESP). Processo n. 2009/50355-4.

**Leia-se:**

## Apoio financeiro

Fundação de Amparo à Pesquisa do Estado de São Paulo (FAPESP). Processo n. 2009/50355-4. O presente trabalho foi realizado com apoio da Coordenação e Aperfeiçoamento de Pessoal de Nível Superior - Brasil (CAPES) - Código de Financiamento 001.
